# Barium and Radium Complexation with Ethylenediaminetetraacetic Acid in Aqueous Alkaline Sodium Chloride Media

**DOI:** 10.1007/s10953-017-0679-7

**Published:** 2017-10-20

**Authors:** Artem V. Matyskin, Niklas L. Hansson, Paul L. Brown, Christian Ekberg

**Affiliations:** 1 Nuclear Chemistry and Industrial Materials Recycling Groups, Energy and Materials Division, Chemistry and Chemical Engineering Department, Chalmers University of Technology, Kemivägen 4, 412 96 Gothenburg, Sweden; 2 Rio Tinto Growth and Innovation, 1 Research Avenue, Bundoora, 3083 VIC Australia

**Keywords:** Alkaline-earth metal, EDTA, Complex formation, Activity coefficient, Specific ion interaction theory, Infinite dilution

## Abstract

The speciation of Ra^2+^ and Ba^2+^ with EDTA was investigated at 25 °C in aqueous alkaline NaCl media as a function of ionic strength (0.2–2.5 mol·L^−1^) in two pH regions where the EDTA^4−^ and HEDTA^3−^ species dominate. The stability constants for the formation of the [BaEDTA]^2−^ and [RaEDTA]^2−^ complexes were determined using an ion exchange method. Barium-133 and radium-226 were used as radiotracers and their concentrations in the aqueous phase were measured using liquid scintillation counting and gamma spectrometry, respectively. The specific ion interaction theory (SIT) was used to account for [NaEDTA]^3−^ and [NaHEDTA]^2−^ complex formation, and used to extrapolate the logarithms of the apparent stability constants (log_10_ *K*) to zero ionic strength (BaEDTA^2−^: 9.86 ± 0.09; RaEDTA^2−^: 9.13 ± 0.07) and obtain the Ba^2+^ and Ra^2+^ ion interaction parameters: [*ε*(Na^+^, BaEDTA^2−^) = − (0.03 ± 0.11); *ε*(Na^+^, RaEDTA^2−^) = − (0.10 ± 0.11)]. It was found that in the pH region where HEDTA^3−^ dominates, the reaction of Ba^2+^ or Ra^2+^ with the HEDTA^3−^ ligand also results in the formation of the BaEDTA^2−^ and RaEDTA^2−^ complexes (as it does in the region where the EDTA^4−^ ligand dominates) with the release of a proton. Comparison of the ion interaction parameters of Ba^2+^ and Ra^2+^ strongly indicates that both metal ions and their EDTA complexes have similar activity coefficients and undergo similar short-range interactions in aqueous NaCl media.

## Introduction

Barium and radium are members of the alkaline-earth metal group. While barium is an abundant element in the earth’s crustal rocks (340 mg·kg^−1^), radium occurs in nature only in trace amounts (0.1 ng·kg^−1^) [1]. Radium has no stable isotopes and the most abundant radium isotope is ^226^Ra with a half-life of 1600 years. Radium-226 is part of the ^238^U decay chain and decays to the short lived (*t*_1/2_ = 3.4 d) α-emitting gas ^222^Rn.

Both ^226^Ra and ^222^Rn are among the most radiotoxic elements present in the environment [2]. As a consequence of some anthropogenic processes, ^226^Ra is concentrated in waste streams. For example, in uranium mining, uranium is usually leached from milled uranium ore or leached in situ using sulfuric acid. After leaching, the tailings (solid and liquid residues) are usually neutralized and disposed in surface ponds in the form of a slurry [3, 4]. Predominantly, radium is rapidly dissolved in leaching and co-precipitates in the form of Ba(Ra)SO_4_ [5]. The concentration of ^226^Ra in such tailings is higher than in the natural uranium ore and can reach up to 43.4 kBq·kg^−1^ (1186.7 ng·kg^−1^) [6]. The background radiation levels are also increased, mostly because of radium and its decay products, for example, from 0.1 to 0.2 μSv·h^−1^ in reference areas such as the tailings storage facility up to 10–20 μSv·h^−1^ on the top of waste dumps [6]. Radium-226 concentrations up to 200 Bq·L^−1^ (0.2 nmol·L^−1^) also occur in water produced from the petroleum industry, which is above limits for industrial effluents [7]. Radium-226 is usually removed by addition of sulfate salts which allow it to co-precipitate in the form of Ba(Ra)SO_4_. Therefore, co-precipitation of radium with barite (BaSO_4_), mostly via an inclusion (lattice replacement) process [7], is the main mechanism controlling radium behavior in the waste streams and its migration in the environment [5, 8]. To decontaminate uranium tailings or solid residues from, e.g., the petroleum industry, it is necessary to dissolve Ba(Ra)SO_4_.

Pure radium and barium sulfate salts and their co-precipitates are, in principle, insoluble in water and aqueous solutions of mineral acids and alkali at room temperature [9] (the recommended values for the decadic logarithm of the BaSO_4_ and RaSO_4_ solubility products at zero ionic strength and 25 °C are −9.95 and −10.21, respectively [10, 11]). At room temperature, Ba(Ra)SO_4_ can be dissolved using chelating agents. The most commercially available chelating agent for Ba(Ra)SO_4_ dissolution is ethylenediaminetetraacetic acid (EDTA) and its derivatives. Aqueous alkaline EDTA solutions have been found to be effective in the dissolution of Ba(Ra)SO_4_ and in the extraction of ^226^Ra from uranium tailings [12]. Approximately 80–85% of ^226^Ra was extracted from uranium tailings using a 0.04 mol·L^−1^ aqueous alkaline EDTA solution at Elliot Lake, Ontario, Canada [13]. Moreover, alkaline EDTA solutions have been used for dissolution of irradiated ^226^RaSO_4_ targets and the preparation of ^227^Ac/^223^Ra radiopharmaceutical generators [14]. One of the reasons for the high Ba(Ra)SO_4_ solubility in alkaline EDTA solutions is the formation of a strong complex between Ba^2+^ or Ra^2+^ and EDTA. Therefore, it is necessary to know accurately the stability constants of the BaEDTA^2−^ and RaEDTA^2−^ complexes to model the Ba(Ra)SO_4_ dissolution equilibrium in alkaline EDTA systems including decontamination using EDTA.

Experimental studies of Ba^2+^ and Ra^2+^ complex formation are also important on a fundamental level. Radium and barium have similar solution chemistry and one of the main reasons for this is the similarity of the effective ionic radii, which are equal to 1.42 Å for Ba^2+^ and 1.48 Å for Ra^2+^ (in 8-fold coordination) [15]. Due to the high radiotoxicity of radium and its daughters, experimental thermodynamic data for radium are limited. For example, to the best of our knowledge, the experimental determination of radium activity coefficients or ion interaction parameters have never been reported in the literature. Due to the lack of experimental data, extrapolation of the ion interaction parameters for radium from values of the other alkaline-earth metals using ionic radii or using interaction parameters of barium directly are the methods used to calculate radium activity coefficients [5, 16, 17]. All approaches for modelling activity coefficients are semi-empirical, with one or more fitted parameters, thus the obtained ion interaction parameters can be brought into question. Therefore, an experimental study of Ba^2+^ and Ra^2+^ complex formation using a background electrolyte would be beneficial on both applied and fundamental levels.

The objective of this work was to study the complex formation of Ra^2+^, as well as Ba^2+^, with EDTA as a function of ionic strength using NaCl as an ionic medium. Sodium chloride is an inert ionic electrolyte which is also omnipresent in the environment. Due to the high radiotoxicity of radium, the complex formation was studied via an ion exchange method which only requires trace amounts of radium. The specific ion interaction theory (SIT) was used to extrapolate the apparent stability constants of the studied complexes to zero ionic strength, and for determining the ion interaction parameters of the species involved in the complex formation.

## Experimental Section

### Sample Preparation

The complexation of Ba^2+^ and Ra^2+^ with EDTA was studied as a function of NaCl ionic strength (0.22, 0.5, 1.0, 2.0 and 2.5 mol·L^−1^) via an ion exchange method with batch and radiotracer techniques. The method is based on the different distribution of metal ions (^133^Ba^2+^ or ^226^Ra^2+^) and negatively charged metal–EDTA complexes using a strong cation exchange resin. Distribution experiments were performed in polypropylene tubes with aqueous phase volumes of 10 mL in the case of Ba^2+^, and 1 mL in the case of Ra^2+^, with 0.5 g (Ba^2+^) and 0.05 g (Ra^2+^) of ion exchange resin added to each tube. The ionic strength in the aqueous phase was adjusted using concentrated NaCl stock solutions. Different doses of Na_2_EDTA stock solution were added to each sample and its concentration was varied throughout the sample series, ranging between 0 and 6.67 × 10^−5^ mol·L^−1^. The apparent EDTA dissociation constants at various NaCl ionic strengths were determined using the SIT methodology and the H^+^ concentration was adjusted using potentiometric titrations to maximize the molar fractions of EDTA^4−^ (−log_10_ [H^+^] = 12.4; more than 99% EDTA^4−^) or HEDTA^3−^ (−log_10_ [H^+^] = 7.9–8.3 depending on the ionic strength; always more than 98% HEDTA^3−^). Samples without the ion exchange resin and EDTA were prepared to measure the total radioactivity of ^133^Ba^2+^ or ^226^Ra^2+^ in the samples. Preliminary kinetic studies confirmed that the metal–EDTA equilibria were achieved within 24 h under the experimental conditions used. The experiments were performed in duplicate where each series contained 11 samples per ionic strength. All samples were kept at 25 ± 1 °C.

### Chemicals Used

All aqueous solutions were prepared using MQ water with 18.2 MΩ·cm resistivity at 25 °C and a total organic content of less than 5 mg·L^−1^. The barium stock solution was in the form of ^133^Ba with a specific activity of 37 kBq·µL^−1^ in 0.1 mol·L^−1^ HCl with an additional 10 µg·mL^−1^ of BaCl_2_ carrier (Eckert and Ziegler Isotope Products radionuclide purity > 99%). Radium carbonate was synthesized from RaSO_4_ powder as previously described [9]. The synthesized RaCO_3_ was dissolved in 0.1 mol·L^−1^ HCl (Sigma–Aldrich 99.999% trace metals basis) to obtain 14 mL of radium stock solution with a ^226^Ra specific activity of (2.5 ± 0.1) × 10^4^ Bq·µL^−1^. The purity of the synthesized radium stock solution was measured previously and it was found that the mass fraction of stable barium and lead was 0.2 and 0.003, respectively [18]. The cation exchange resin was in sodium form (Biorad AG 50W-X8 200–400 mesh molecular biology grade). EDTA stock solutions were prepared from solid Na_2_EDTA·2H_2_O (Sigma p.a. ≥ 99.0%). The ionic strength and −log_10_ [H^+^] were adjusted using a NaCl stock solution prepared from solid NaCl (Sigma–Aldrich ACS reagent p.a. ≥ 99.0%) and standard NaOH and HCl solutions (Fixanal, Sigma-Aldrich).

### Apparatus

All solid chemicals were weighed on a standard analytical balance (Sartorius Quintix125D-1S) and samples were kept at a constant temperature of 25 ± 1 °C in a shaking water bath (Julabo SW23). Potentiometric measurements were performed using two pH meters coupled with combined glass electrodes (827 pH laboratory Metrohm coupled with Metrohm Primatrode electrode and Radiometer MeterLab PHM240 coupled with A Radiometer PHC3006-9 electrode). Both electrodes were filled with a 3 mol·L^−1^ NaCl reference electrolyte and calibrated using the activity scale with standard buffer solutions (NIST and SRM traceable, Certipur, Merck), and were subsequently calibrated in the concentration scale using a potentiometric titration with negligible volume change [19]. The radioactivity of ^133^Ba was measured using liquid scintillation counting (LSC) (Perkin Elmer Guardian 1414) and aqueous ^133^Ba samples were subsequently mixed with an Emulsifier safe LSC cocktail. The radioactivity of ^226^Ra was measured using two High Purity Germanium detectors (HPGe) (Canberra GEM23195 closed-end coaxial HPGe detector coupled with digital spectrum analyzer Canberra-2000/A and Ortec GEM-C5060 coaxial HPGe coupled with digital spectrum analyzer Ortec DSPEC50). Both detectors were calibrated using a mixed radionuclide reference solution (NIST traceable, Eckert and Ziegler). Nuclide half-lives, gamma emission energies and photon emission probabilities were taken from the Decay Data Evaluation Project [20].

## The Model

The speciation of a metal ion (M^2+^) with various forms of EDTA can be described by the reaction:

1 $$ {\text{M}}^{2 + } + [{\text{H}}_{r} {\text{EDTA}}^{ (r - 4 )} ] \rightleftharpoons [{\text{MH}}_{r} {\text{EDTA}}^{ (r - 2 )} ] $$

where 0 ≤ *r* ≤ 6.

The stability constant for reaction 1 at zero ionic strength is defined as:

2 $$ K_{{{\text{MH}}_{r} {\text{EDTA}}^{ (r - 2 )} }}^{\text{o}} = K_{{{\text{MH}}_{r} {\text{EDTA}}^{ (r - 2 )} }} \cdot \frac{{\gamma_{{{\text{MH}}_{r} {\text{EDTA}}^{ (r - 2 )} }} }}{{\gamma_{{{\text{M}}^{2 + } }} \cdot \gamma_{{{\text{H}}_{r} {\text{EDTA}}^{ (r - 4 )} }} }} = \frac{{[{\text{MH}}_{r} {\text{EDTA}}^{ (r - 2 )} ]}}{{[{\text{M}}^{2 + } ] \cdot [{\text{H}}_{r} {\text{EDTA}}^{ (r - 4 )} ]}} \cdot \frac{{\gamma_{{{\text{MH}}_{r} {\text{EDTA}}^{ (r - 2 )} }} }}{{\gamma_{{{\text{M}}^{2 + } }} \cdot \gamma_{{{\text{H}}_{r} {\text{EDTA}}^{ (r - 4 )} }} }} $$

The SIT model developed by Brønsted [21, 22], Scatchard [23], Guggenheim and Turgeon [24] can be used to express the activity coefficients *γ*_*i*_ of an ion *i* at ionic strengths below about 3.5 mol·kg^−1^:

3 $$ \log_{10} \,\gamma_{i} = -\, z_{i}^{2} \cdot D_{\text{H}} + \sum\limits_{j} {\varepsilon (i,j,I_{m} )} \cdot m_{j} $$

where *z*_*i*_ is the charge of the ion *i*, *ε(i,j,I*_*m*_ *)* is the interaction parameter of ion *i* with all oppositely charged ions *j*, *I*_*m*_ is ionic strength in mol·kg^−1^, *m*_*j*_ is molal concentration of ion *j* and *D*_H_ is the Debye–Hückel term which is defined as:

4 $$ D_{\text{H}} = \frac{{A \cdot \sqrt {I_{m} } }}{{1 + 1.5 \cdot \sqrt {I_{m} } }} $$

where *A* is a temperature dependent constant equal to 0.5090 and 0.5047 kg^1/2^·mol^−1/2^ at 25 °C and 20 °C, respectively, for aqueous solutions [25]. The value 1.5 is the product of *B* (a constant dependent on temperature and the solvent relative permittivity) and *a* (distance of closest approach or effective Debye–Hückel ionic radius). In the SIT, this product is usually taken to be 1.5 to minimize the effect of ionic strength on the ion interaction parameters. In this work, each ionic strength of NaCl was recalculated to the molal scale (from molar) using the relevant conversion factors [25]. Substituting the activity coefficients calculated using Eq. 3 into Eq. 2 yields:

5 $$ \log_{10} \,K_{{{\text{MH}}_{r} {\text{EDTA}}^{ (r - 2 )} }} - \Delta z^{2} \cdot D_{\text{H}} = \log_{10} \,K_{{{\text{MH}}_{r} {\text{EDTA}}^{ (r - 2 )} }}^{\text{o}} - \Delta \varepsilon \cdot I_{m} $$

From Eq. 5 it can be concluded that plotting the difference between the determined decadic logarithm of the apparent stability constants and ∆*z* ^2^·*D*_H_ against ionic strength of the same background electrolyte will result in an intercept which is the decadic logarithm of the stability constant at zero ionic strength and a slope which is the ion interaction parameter term.

Measurement of the metal ion radioactivity in the aqueous phase allows for calculation of the distribution ratio between the solid phase and the aqueous phase according to:

6 $$ D = \left( {\frac{{A_{\text{total}} - A_{\text{aq}} }}{{A_{\text{aq}} }}} \right) \cdot \frac{V}{m} $$

where *A*_total_ is the total radioactivity of the metal ion in the sample, *A*_aq_ is the radioactivity of the metal ion in the aqueous phase after the distribution equilibrium has been reached, *V* is the solution volume (mL) and *m* is the mass of the ion exchange resin (g).

The distribution ratio can also be expressed through the apparent stability constant:

7 $$ D = \frac{{\lambda \cdot [{\text{M}}^{2 + } ]}}{{[{\text{M}}^{2 + } ] + \sum {(K_{{{\text{MH}}_{r} {\text{EDTA}}^{ (r - 2 )} }} \cdot [{\text{M}}^{2 + } ] \cdot [{\text{H}}_{r} {\text{EDTA}}^{ (r - 4 )} ])} }} $$

where *λ* is the distribution ratio without the ligand (mL·g^−1^) and *K* is the apparent stability constant for the MH_*r*_EDTA^(*r*−2)^ complex.

The apparent dissociation constants of the H_r_EDTA^(*r*−4)^ complexes can be computed via the SIT (Eq. 3) using the EDTA dissociation constants at zero ionic strength and their ion interaction parameters given in the literature [26]. The constants calculated in this manner have been used in this work. Molar fractions of the different EDTA species can be computed as a function of hydrogen ion concentration using the calculated apparent dissociation constants of H_*r*_EDTA^(*r*−4)^. The concentration of H^+^ at which the molar fractions of EDTA^4−^ and HEDTA^3−^ are maximized were calculated for all studied ionic strengths, and −log_10_ [H^+^] was adjusted according to these calculations.

The hydrolysis of Ba^2+^ and Ra^2+^ at a −log_10_ [H^+^] of 12.4 (the highest −log_10_ [H^+^] used in this work) can be neglected [27] compared to the metals strong complexation with EDTA. Polynuclear complexes are also not formed when a metal ion is at radiotracer levels, therefore the M^2+^ concentration terms in Eq. 7 cancel. Only one form of H_*r*_EDTA^(*r*−4)^ is dominant under each of the two experimental conditions studied. As a result, Eq. 7 can be simplified to:

8 $$ K_{{{\text{MH}}_{r} {\text{EDTA}}^{ (r - 2 )} }} \cdot [{\text{H}}_{r} {\text{EDTA}}^{ (r - 4 )} ] = \frac{\lambda }{D} - 1 $$

Thus, the apparent stability constants of the MH_*r*_EDTA^(*r*−2)^ complexes can be determined using linear regression.

The [H_*r*_EDTA^(*r*−4)^] term in Eq. 8 refers to the *free* concentration of the ligand. However, EDTA also forms strong complexes with Na^+^, which was used as part of the ionic medium. The Na^+^ concentration was considerably higher than the M^2+^ concentration under all experimental conditions. As a result, the concentration of *free* EDTA was adjusted by the EDTA complex formation with Na^+^. The effect of complex formation between EDTA^4−^ or HEDTA^3−^ and Na^+^ has been found to be important [28] and can be described by the following reactions:

9 $$ {\text{Na}}^{ + } + {\text{EDTA}}^{4 - } \rightleftharpoons {\text{NaEDTA}}^{3 - } $$

10 $$ {\text{Na}}^{ + } + {\text{HEDTA}}^{3 - } \rightleftharpoons {\text{NaHEDTA}}^{2 - } $$

As a result, the *free* EDTA^4−^ or HEDTA^3−^ concentration in Eq. 8 can be expressed as:

11 $$ [{\text{EDTA}}_{\text{free}}^{4 - } ] = \frac{{[{\text{EDTA}}_{\text{total}}^{ 4- } ]}}{{1 + K_{{}}^{\text{HEDTA}} \cdot [{\text{H}}^{ + } ] + K_{{}}^{\text{NaEDTA}} \cdot [{\text{Na}}^{ + } ]}} $$

12 $$ [{\text{HEDTA}}_{\text{free}}^{ 3- } ] = \frac{{[{\text{EDTA}}_{\text{total}}^{ 4- } ]}}{{1 + \frac{{[{\text{H}}^{ + } ]}}{{K_{{}}^{\text{HEDTA}} }} + K_{{}}^{\text{NaHEDTA}} \cdot [{\text{Na}}^{ + } ]}} $$

where *K* ^HEDTA^ refers to the protonation constant of EDTA^4−^ and *K* ^NaEDTA^ or *K* ^NaHEDTA^ refer to the stability constants for reactions 9 and 10, respectively.

## Results and Discussion

### Sodium Speciation with EDTA

The dissociation constant of EDTA and stability constant for reaction 9 have been experimentally studied by many researchers and a comprehensive review is available [26]. The values of the protonation constants and the NaEDTA^3−^ stability constant at zero ionic strength were taken from Hummel and co-workers [25] and are listed in Table 1. The SIT ion interaction parameters and associated uncertainties were derived from all available experimental data of NaEDTA^3−^ and EDTA^4−^ protonation in NaCl media at 25 °C listed in the review [26]. Subsequently, the apparent stability constants were calculated using the derived SIT ion interaction parameters. The apparent EDTA^4−^ protonation constants and NaEDTA^3−^ stability constants obtained were used to calculate the Ba^2+^ and Ra^2+^ stability constants (see Table 5) and free EDTA^4−^ concentration (Eq. 11), respectively. All these stability constants are listed in Table 1.

**Table 1:** Stability constants and SIT ion interaction parameters at 25 °C used in this work

Equilibrium reaction	*I*_*m*_ (mol·kg^−1^)	Stability constant log_10_ *K*	Specific ion interaction parameters (NaCl) ∆*ε* (mol·kg^−1^)
H^+^ + HEDTA^3−^ ⇌ H_2_EDTA^2−^	0	6.80 ± 0.02	0.40 ± 0.03
H^+^ + EDTA^4−^ ⇌ HEDTA^3−^	0	11.24 ± 0.03	0.55 ± 0.04
	0.22	10.24 ± 0.03	
	0.51	10.12 ± 0.03	
	1.02	10.21 ± 0.04	
	2.09	10.51 ± 0.06	
	2.64	10.74 ± 0.08	
Na^+^ + EDTA^4−^ ⇌ NaEDTA^3−^	0	2.80 ± 0.20	0.27 ± 0.33
	0.22	1.74 ± 0.22	
	0.51	1.54 ± 0.31	
	1.02	1.44 ± 0.52	
	2.09	1.51 ± 1.0	
	2.64	1.59 ± 1.3	

Only a few experimental data for the formation of the NaHEDTA^2−^ complex (Eq. 10) are available in the literature and the reported log_10_ *K*° values vary significantly from 0 to 1.5 [29–32]. The main reason for the log_10_ *K*° data discrepancies is that the NaHEDTA^2−^ complex is quite weak. In the case of weak complex formation, it is usually impossible to separate the weak complex formation effect from potential activity coefficient changes. This and other challenges associated with the determination of the stability constants of weak complexes have been previously discussed in detail [33, 34]. Perhaps, the most reasonable value for the stability constant of the NaHEDTA^2−^ complex was reported by Palaty [31]. The author used ion selective electrodes to study the proton dissociation reactions of EDTA and the sodium–EDTA equilibrium and the obtained stability constant values are in good agreement with the values listed in Table 1 (11.34, 6.81 and 2.61, respectively [31]). Tetramethylammonium chloride was used as the background electrolyte with a total ionic strength of 0.12 mol·L^−1^. The temperature was not given by the author [31] but based on all the obtained values it can be assumed that the reported equilibria were studied at 25 °C. The reported value for the log_10_ *K*° value of the NaHEDTA^2−^ complex was −0.03. The value is subject to some uncertainty and it is assumed that the actual log_10_ *K*° value at zero ionic strength lies in the range from −0.5 to 0.5 (i.e., log_10_ *K* = 0 ± 0.5). Most probably, the assignment of such a high, but reasonable, uncertainty for the stability constant of a weak complex is the only way to overcome the lack of reliable data. The proposed log_10_ *K*° value of 0 ± 0.5 is in accord with the statement made by Marcus and Hefter in relation to log_10_ *K*° values less than 1, where substantial care needs to be taken in obtaining the exact magnitude of such constants by either experiment or theory [34].

To be able to extrapolate the log_10_ *K*° value of 0 ± 0.5 for the NaHEDTA^2−^ complex at the ionic strengths used in this work, it is necessary to know the following SIT interaction parameters: *ε*(Na^+^, Cl^−^), *ε*(Na^+^, HEDTA^3−^) and *ε*(Na^+^, NaHEDTA^2−^). The first two parameters, with their associated uncertainties, are available in the literature [25, 26] and to the best of our knowledge the last parameter has never been reported. A comparison of the sodium SIT ion interactions with many different negatively charged ligands shows that this parameter usually varies from −0.3 to 0.1 [25] (the sodium ion with a divalent anion). Moreover, the sodium SIT ion interaction with ligands similar to H_2_EDTA^2−^ is −0.37 [26]. Consequently, based on these values, the *ε*(Na^+^, NaHEDTA^2−^) SIT parameter has been estimated as −(0.2 ± 0.3) kg·mol^−1^. All the parameters associated with the NaHEDTA^2−^ complex (Eq. 10) used in this work are listed in Table 2.

**Table 2:** Stability constants and SIT ion interaction parameters for the NaHEDTA^2−^ complex formation (Eq. 2) at 25 °C

Parameter	Value	References
log_10_ *K*°	0 ± 0.5	Estimated in this work, based on available experimental data from Palaty [31]
*ε*(Na^+^, Cl^−^)	0.03 ± 0.01 (kg·mol^−1^)	Guillaumont et al. [25]
*ε*(Na^+^, HEDTA^3−^)	−(0.1 ± 0.14) (kg·mol^−1^)	Hummel et al. [26]
*ε*(Na^+^, NaHEDTA^2−^)	−(0.2 ± 0.3) (kg·mol^−1^)	Estimated in this work

### Stability Constants for the Complex Formation of Ba^2+^ and Ra^2+^ with EDTA

The apparent stability constants for the BaEDTA^2−^ and RaEDTA^2−^ complexes were obtained from distribution coefficients (from experiments conducted at a −log_10_ [H^+^] of 12.4) using a weighted linear regression (*ω*_*i*_ = *σ*_*i*_) with a zero intercept (Eq. 8). The free EDTA^4−^ concentrations were obtained by correcting for the formation of the NaEDTA^3−^ complex (Eq. 9) using Eq. 11 and the values which are listed in Table 1. The standard deviations of the free EDTA^4−^ concentrations were propagated from the standard deviation of the apparent NaEDTA^3−^ stability constants, also listed in Table 1. The standard deviations of the distribution ratio without the ligand (*λ*) and the distribution ratio with the ligand (*D*) were calculated based on duplicate series (biased standard deviation with (*n* − 1) in the denominator) and were propagated to the standard deviations of (*λ*/*D* − 1). Standard uncertainty propagation was used in the both cases.

The uncertainties in the linear fitting were obtained using the method of Allard and Ekberg [35]. After obtaining the uncertainties in both the (*λ*/*D* − 1) term and the free EDTA concentration, 30 points were sampled from each uncertainty space using a normal distribution with the mean and standard deviation obtained. Thus, the obtained simulated data points covered the entire standard deviation region in both *x* and *y* forming confidence ellipses for each point. Negative simulated values of the free EDTA^4−^ concentrations were discarded. All these points were then used for the linear regression and the estimation of the associated uncertainty analysis.

**Fig. 1:**
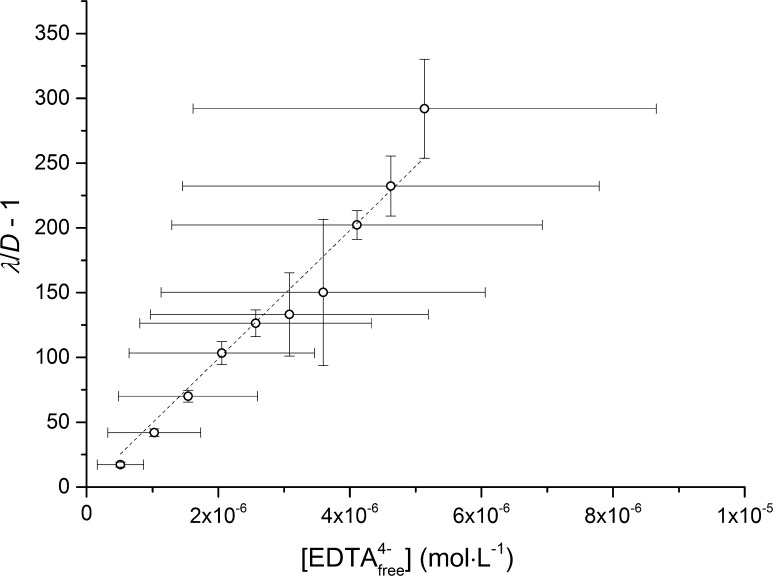
Determination of BaEDTA^2−^ apparent stability constants using linear regression (0.22 mol*·*kg^−1^ NaCl, reaction 1, Eq. 8)

Figure 1 shows a representative dataset for the linear regression of the BaEDTA^2−^ (reaction 1) apparent stability constant in 0.22 mol·kg^−1^ NaCl.

As can be observed from Fig. 1, the standard deviations of the free EDTA^4−^ concentrations are large and increase with an increase in ionic strength (NaCl). These large standard deviations are a consequence of the error propagation that results principally from the large uncertainties in the NaEDTA^3−^ stability constants (Table 1).

**Fig. 2:**
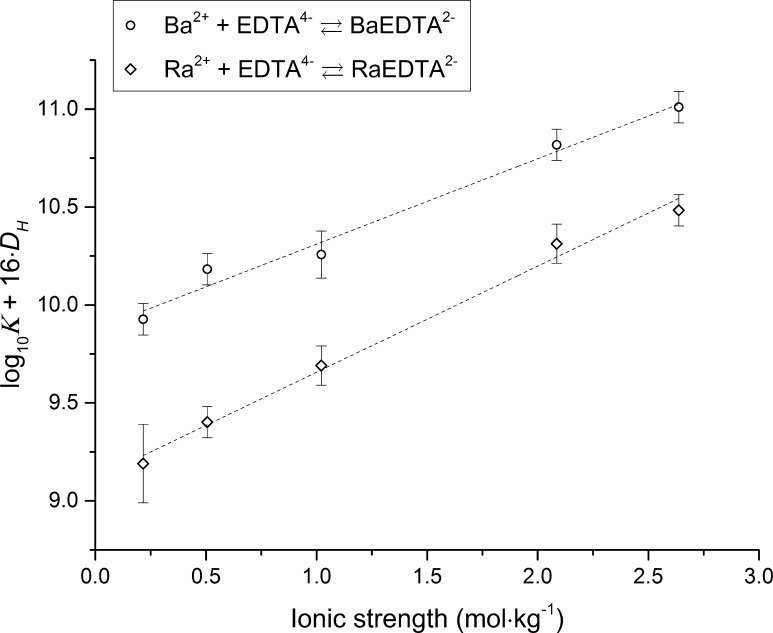
Extrapolation of BaEDTA^2−^ and RaEDTA^2−^ apparent stability constants (NaCl media, reaction 1) to zero ionic strength using SIT

The stability constants obtained are listed in Table 3 and extrapolation of the BaEDTA^2−^ and RaEDTA^2−^ stability constants to zero ionic strength (non-weighted linear regression) using the SIT are shown in Fig. 2.

**Table 3:** Apparent stability constants of BaEDTA^2−^ and RaEDTA^2−^ aqueous complexes in NaCl media at 25 °C formed via reaction 1

*I*_*m*_ (mol·kg^−1^)	log_10_ *K*_BaEDTA_	log_10_ *K*_RaEDTA_
0	9.88 ± 0.11	9.11 ± 0.09
0.22	7.70 ± 0.08	6.96 ± 0.20^a^
0.51	7.38 ± 0.08	6.60 ± 0.08
1.02	6.99 ± 0.12	6.42 ± 0.10
2.09	7.10 ± 0.08	6.60 ± 0.10
2.64	7.16 ± 0.08	6.63 ± 0.08

Ionic strengths were adjusted from the mol·L^−1^ to mol·kg^−1^ scale using the appropriate conversion factors [25]. Uncertainties correspond to 95% confidence intervals

^a^Estimated uncertainty

As can be observed from Fig. 2, the fits are satisfactory and the experimental data are accurately modelled by the SIT. According to the calculations, the effect of Na^+^ complex formation with EDTA^4−^ (Eq. 9) is significant and the difference between the corrected and uncorrected stability constants of both BaEDTA^2−^ and RaEDTA^2−^ at zero ionic strength is more than 1 log_10_ unit. The difference between the slopes (with and without correction for Na complex formation with EDTA), which corresponds to the ion interaction parameter term, was also significant and the deviation of the experimental data points from the regression line was higher at increased ionic strength. This strongly indicates that the complex formation between sodium and EDTA is significant, which is in agreement with previous studies [28].

**Fig. 3:**
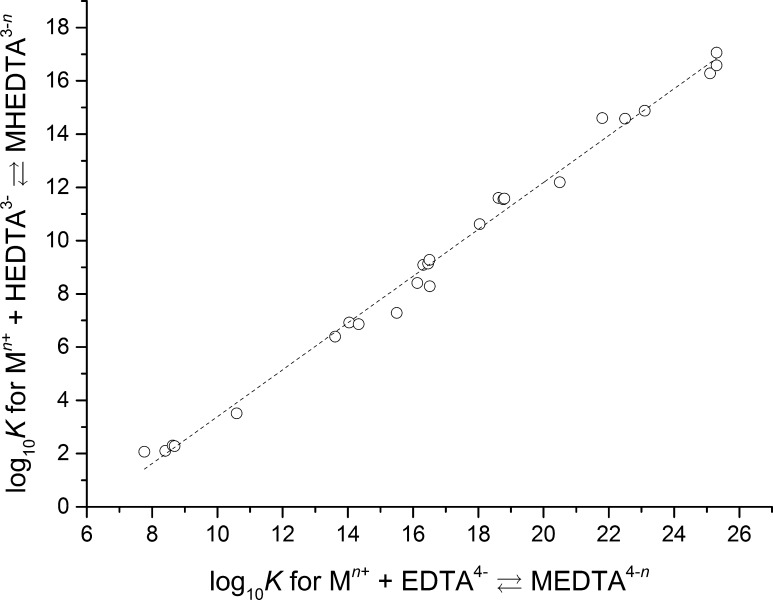
Linear free energy analysis of available literature data [36–38] for the decadic logarithm of M^*n*+^EDTA^(4−*n*)^ and M^*n*+^HEDTA^(3−*n*)^ apparent stability constants (*n* ≥ 2) at the same experimental conditions (20 °C, *I* = 0.1 mol·L^−1^)

The apparent stability constants, assuming only the formation of the BaHEDTA^−^ and RaHEDTA^−^ complexes [according to reaction 1 (*r* = 1)], were derived from the experiments conducted at −log_10_ [H^+^] of 7.9–8.3 with the mole fraction of HEDTA^3−^ being more than 98% using the same method as used for derivation of the BaEDTA^2−^ and RaEDTA^2−^ complex stability constants. The apparent stability constants obtained were extrapolated to zero ionic strength using the SIT that resulted in stability constants of log_10_ *K°* = 7.34 ± 0.30 (for BaHEDTA^−^) and log_10_ *K*° = 6.57 ± 0.30 (for RaHEDTA^−^). Schwarzenbach and Ackermann [36] have previously given a log_10_ *K* value for the same reaction (BaHEDTA^−^ complex) of 2.07 at 20 °C and an ionic strength of 0.1 mol·L^−1^. This value, when extrapolated to zero ionic strength, results in log_10_ *K*° = 3.15, which is much lower than the value obtained in the present work. It can be seen that the value from this study is more than four orders of magnitude larger than the value given by Schwarzenbach and Ackermann. There are two probable reasons for the disagreement between these two values: either the assumption that the BaHEDTA^−^ complex is formed according to reaction 1 (*r* = 1) at −log_10_ [H^+^] of 7.9–8.3 is not valid or the data from Schwarzenbach and Ackermann are inconsistent. The latest hypothesis can be verified by combining the data from Schwarzenbach and Ackermann [36] with other literature data [37, 38], where the stability constants for the reaction of various metals with EDTA^4−^ and HEDTA^3−^ are reported for the same experimental conditions (20 °C and an ionic strength of 0.1 mol·L^−1^) and performing a linear free energy analysis of the data. This analysis (i.e., a plot of the log_10_ *K* values of M^*n*+^–EDTA^4−^ complexes against the log_10_ *K* of M^*n*+^–HEDTA^3−^ complexes, where M^*n*+^ is a metal ion with *n* ≥ 2 (reaction 1 with *r* = 0 and *r* = 1, respectively)) is shown in Fig. 3.

As shown in Fig. 3, there is a strong relationship between the magnitude (log_10_ *K* values) of the M^*n*+^EDTA^(4−*n*)^ and M^*n*+^HEDTA^(3−*n*)^ stability constants (*n* ≥ 2), and consequently, the available literature data [36–38] are consistent. Therefore, the assumption that only the BaHEDTA^−^ or RaHEDTA^−^ complexes are formed at a −log_10_ [H^+^] of 7.9–8.3 is not valid. The stability constant for the BaHEDTA^−^ complex derived in the present study is more than four orders of magnitude larger when compared to those values available in the literature, which indicates that another stronger complex dominates at a −log_10_ [H^+^] of 7.9–8.3. The only other strong complex that could be formed in the studied system is BaEDTA^2−^ (or RaEDTA^2−^). The likely mechanism of the formation of these two complexes at a −log_10_ [H^+^] of 7.9–8.3, where the mole fraction of HEDTA^3−^ is more than 98% is as follows:

13 $$ {\text{Ba}}^{2 + } + {\text{HEDTA}}^{3 - } \rightleftharpoons {\text{BaEDTA}}^{2 - } + {\text{H}}^{ + } $$

14 $$ {\text{Ra}}^{2 + } + {\text{HEDTA}}^{3 - } \rightleftharpoons {\text{RaEDTA}}^{2 - } + {\text{H}}^{ + } $$

If the proposed reactions 13 and 14 occur in the studied system, then Eq. 7 can be adapted to reactions 13 and 14 to describe the experimental data obtained at a −log_10_ [H^+^] of 7.9–8.3:

15 $$ K_{{{\text{M}}_{r} {\text{EDTA}}^{ (r - 4 )} }} \cdot \frac{{[{\text{HEDTA}}^{3 - } ]}}{{[{\text{H}}^{ + } ]}} = \frac{\lambda }{D} - 1 $$

**Fig. 4:**
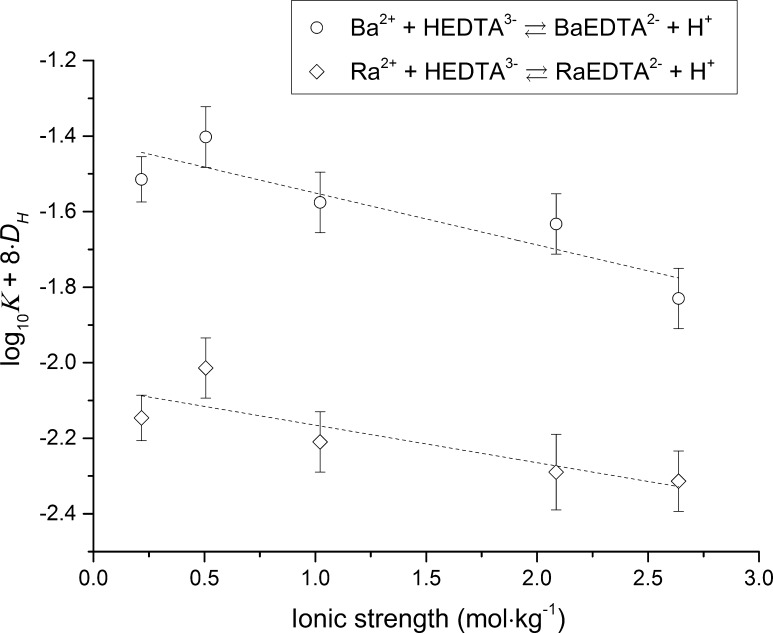
Extrapolation of BaEDTA^2−^ and RaEDTA^2−^ apparent stability constants (NaCl media, reactions 13 and 14) to zero ionic strength using the SIT

According to Eq. 15, the concentration of the free HEDTA^3−^ must be divided by the H^+^ concentration to obtain the apparent stability constant for the BaEDTA^2−^ or RaEDTA^2−^ complex via reactions 13 and 14 under these lower −log_10_ [H^+^] conditions. Moreover, it can be shown that the sum of the decadic logarithm of obtained stability constants for reactions 13 and 14 and the decadic logarithm of the protonation constant of EDTA^4−^ results in the decadic logarithm of the stability constant for the BaEDTA^2−^ or RaEDTA^2−^ complexes formed via reaction 1 with *r* = 0. The stability constants for reactions 13 and 14 at a −log_10_ [H^+^] of 7.9–8.3 and the associated standard deviations were derived using the same method as was used to derive stability constants and standard deviations for reaction 1 with *r* = 0 at a −log_10_ [H^+^] of 12.4. These stability constants and the calculated stability constants for reaction 1 with *r* = 0, using the derived constants and the protonation constants of EDTA^4−^ from Table 1, are listed in Table 4. Extrapolation of the BaEDTA^2−^ and RaEDTA^2−^ stability constants to zero ionic strength using the SIT is shown in Fig. 4.

**Table 4:** Apparent stability constants of BaEDTA^2−^ and RaEDTA^2−^ aqueous complexes in NaCl media at 25 °C formed via reactions 13 and 14 and 1

*I*_*m*_ (mol·kg^−1^)	log_10_ *K*_BaEDTA_ (formed via reaction 13)	log_10_ *K*_BaEDTA_ (formed via reaction 1)	log_10_ *K*_RaEDTA_ (formed via reaction 14)	log_10_ *K*_RaEDTA_ (formed via reaction 1)
0	−1.41 ± 0.12	9.83 ± 0.14	−2.07 ± 0.11	9.17 ± 0.13
0.22	−2.63 ± 0.06	7.61 ± 0.08	−3.26 ± 0.06	6.98 ± 0.08
0.51	−2.80 ± 0.08	7.32 ± 0.10	−3.42 ± 0.08	6.71 ± 0.10
1.02	−3.21 ± 0.08	7.00 ± 0.11	−3.85 ± 0.08	6.37 ± 0.11
2.09	−3.49 ± 0.08	7.02 ± 0.14	−4.15 ± 0.10	6.36 ± 0.15
2.64	−3.75 ± 0.08	6.99 ± 0.18	−4.24 ± 0.08	6.50 ± 0.18

Ionic strengths were adjusted from the mol·L^−1^ to mol·kg^−1^ scale using the appropriate conversion factors [25] and log_10_ *K*_BaEDTA_ or log_10_ *K*_RaEDTA_ for the reactions 13 and 14 were calculated using EDTA^4−^ protonation constants listed in Table 1. Uncertainties correspond to 95% confidence interval

As can be observed in Fig. 4, the experimental data are accurately described by Eq. 15. A comparison of the stability constants of the BaEDTA^2−^ and RaEDTA^2−^ complexes formed via reaction 1 listed in Table 4 with the same stability constants listed in Table 3 shows that all the values are within the 95% confidence intervals. This strongly indicates that the proposed reactions 13 and 14 occur at the pH region where the HEDTA^3−^ species dominates. The effect of Na^+^ complex formation with HEDTA^3−^ (Eq. 10) was not as significant as in the case of EDTA^4−^ due to the fact that the NaHEDTA^2−^ complex is much weaker than NaEDTA^3−^ (Tables 1, 2).

A comparison of the average value of the obtained metal–EDTA stability constants at zero ionic strength with data available in the literature is shown in Table 5. The data from the literature were, where necessary, extrapolated to zero ionic strength using the Davies equation [39] (in the last term 0.2*·I* was used instead of 0.3*·I*, the latter as proposed by Davies [40]) for activity coefficient corrections. The weighted mean and associated 95% confidence intervals of the BaEDTA^2−^ and RaEDTA^2−^ stability constants at zero ionic strength were calculated from the values listed in Tables 3 and 4.

**Table 5:** Comparison of reported stability constants for the formation of BaEDTA^2−^ and RaEDTA^2−^

Method	Ionic medium	Temperature (°C)	Reported log_10_ *K*	Extrapolated to zero ionic strength log_10_ *K*°	Reference
$$\bf{{\textbf{Ba}}^{2+} + {\textbf{EDTA}}^{ 4- } \leftrightharpoons {\textbf{BaEDTA}}^{ 2- } }$$					
Ion exchange	0.2; 0.5; 1.0; 2.0; 2.5 mol·L^−1^ (NaCl)	25	Tables 3 and 4	9.86 ± 0.09	This work
Review	0.1 mol·L^−1^	25	7.86 ± 0.08	9.64	Smith and Martell [38]
pH	0.1 mol·L^−1^ (KCl)	20	7.76	9.54	Schwarzenbach and Ackermann [36]
pH	0.1 mol·L^−1 a^	25	7.73	9.51	Carini and Martell [41]
pH	0.1 mol·L^−1^	25	7.9	9.68	Schmid and Reilley [42]
Ion exchange	0	25	9.92	9.92	Astakhov and Fomenko [43]
pH	0.1 mol·L^−1^ (KNO_3_)	25	7.63	9.41	Bohigian and Martell [44]
Paper electrophoresis	0.1 mol·L^−1^ (KNO_3_)	20	8	9.78	Jokl and Majer [45]
pH	0.1 mol·L^−1^ (KNO_3_ or (CH_3_)_4_N(NO_3_))	25	7.8	9.58	Delgado and Da Silva [46]
$$\bf{{\textbf{Ra}}^{2+} + {\textbf{EDTA}}^{ 4- } \leftrightharpoons {\textbf{RaEDTA}}^{ 2- } }$$					
Ion exchange	0.2; 0.5; 1; 2; 2.5 mol·L^−1^ (NaCl)	25	Table 3	9.13 ± 0.07	This work
Ion exchange	0.1 mol·L^−1 a^	20^a^	7.12	8.9	Nikolsky et al. [47]
Ion exchange	0.1 mol·L^−1 b^ (sodium salt)	20	7.07^b^ ± 0.06	9.22^b^	Baetsle and Bengsch [48]
Solvent extraction	0.1 mol·L^−1^ (NaClO_4_)	25	7.7	9.29	Sekine et al. [49]
Estimated	0.1 mol·L^−1^	25	7.4	9.2	Nelson et al. [50]

^a^Ionic strength and temperature were assumed

^b^Contribution of the 0.01 mol·L^−1^ EDTA to the total ionic strength has been considered

Experimental data for the stability constant of BaEDTA^2−^ [36, 41–46] and reviews of relevant stability constants [38, 51] are available in the literature. The data given in Table 5 for extrapolation of the literature data for the stability constant of BaEDTA^2−^ to zero ionic strength are in very good agreement with the value determined in the present work.

The complex formation of radium with EDTA has been studied by several researchers using the ion exchange or solvent extraction methods and the experimental data have been reviewed [51, 52]. Nikolsky and co-workers were the first to study RaEDTA^2−^ complex formation and obtained a log_10_ *K* value of 7.12 for RaEDTA^2−^ [47]. The value was extrapolated to zero ionic strength assuming a temperature of 20 °C and an ionic strength of 0.1 mol·L^−1^. Baetsle and Bengsch studied RaEDTA^2−^ complex formation using an ion exchange resin (Amberlite IR120) at 20 °C and an ionic strength of 0.1 mol·L^−1^ (sodium salt) and reported a log_10_ *K* value of 7.07 ± 0.06 [48]. The concentration of EDTA^4−^ was 0.01 mol·L^−1^ and an acetate buffer was used. Such a high concentration of EDTA^4−^ has a significant influence on the ionic strength, and therefore, the actual ionic strength used was 0.19 mol·L^−1^ and this value has been used to extrapolate the reported value to zero ionic strength. Sekine and co-workers used solvent extraction (a mixture of 0.1 mol·L^−1^ thenoyltrifluoroacetone and 0.1 mol·L^−1^ tributylphosphate in CCl_4_) to study Ra^2+^ complex formation with various amino carboxylic acids at 25 °C and 0.1 mol·L^−1^ NaClO_4_ and obtained a log_10_ *K* value of 7.7 for the RaEDTA^2−^ complex [49]. A log_10_ *K* value for RaEDTA^2−^ was also estimated to be 7.4 for 25 °C and an ionic strength of 0.1 mol·L^−1^ by Nelson and co-workers [50]. The RaEDTA^2−^ stability constant obtained in this work is in very good agreement with those of the other studies when taking into account differences in temperature, ionic strength and difficulties in analyzing the literature data (experimental details missing, high EDTA concentrations affecting the ionic media etc.). Probably the best comparison of the RaEDTA^2−^ stability constants obtained in this work is with work of Sekine and co-workers and values obtained for zero ionic strength from the two studies are in very good agreement.

**Fig. 5:**
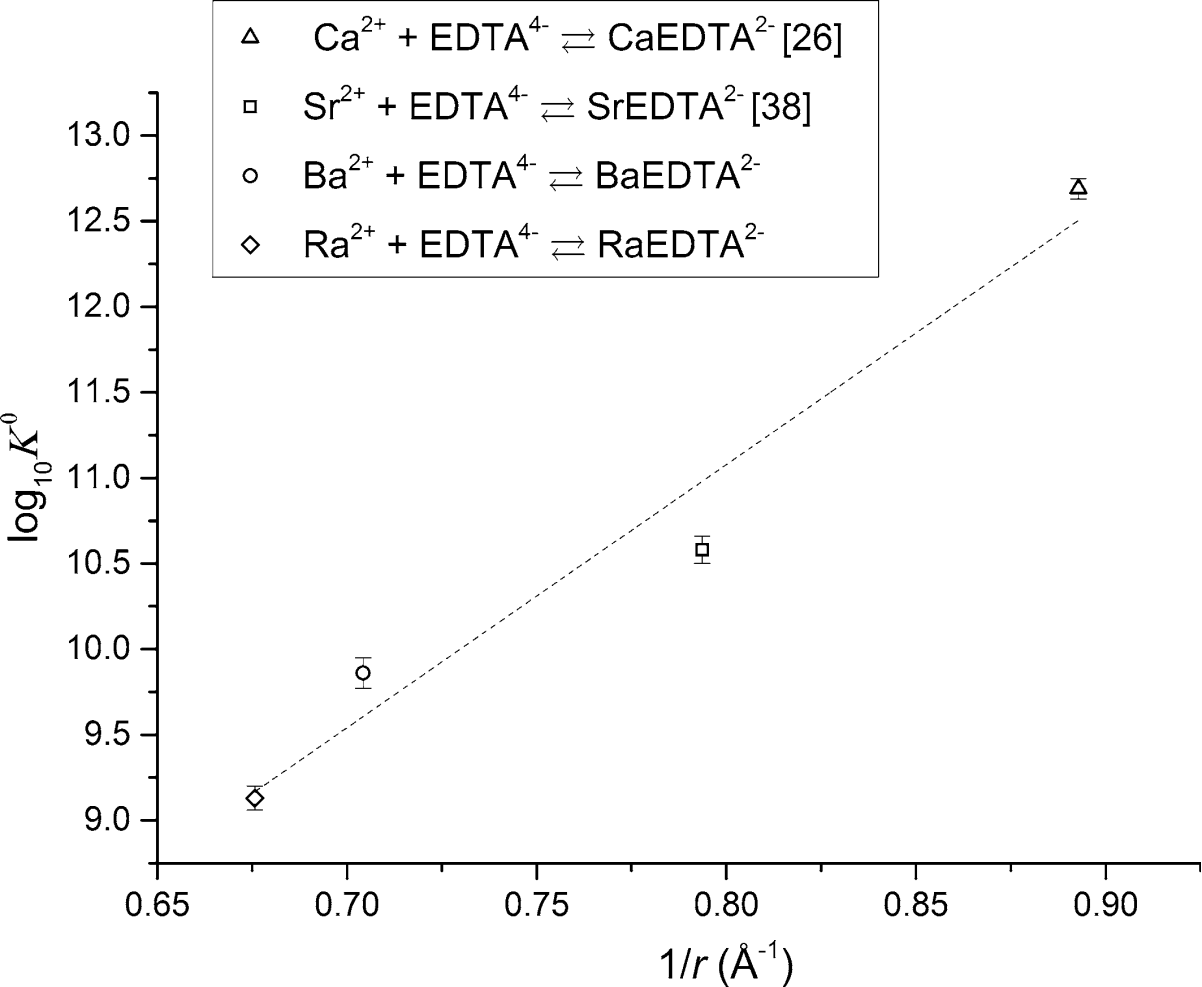
Comparison of alkaline-earth metal–EDTA^4−^ stability constants at zero ionic strength using their effective ionic radii in 8-fold coordination (ionic radii taken from Shannon [15])

The difference between log_10_ *K*°_BaEDTA_^2−^ and log_10_ *K*°_RaEDTA_^2−^ is 0.73 log_10_ units. The difference is relatively small which may indicate that the speciation of Ba^2+^, Ra^2+^, and potentially other alkaline earth metals with EDTA^4−^, depends on the ionic radius of the metal ion. Extrapolation of the thermodynamic properties of radium, including stability constants, from the property values of other alkaline-earth metals using an electrostatic model is a widely used method [8]. A plot of the decadic logarithm of stability constants of calcium (taken from [26]), strontium (taken from [38] and extrapolated to zero ionic strength using the Davies equation), barium and radium with EDTA^4−^ at zero ionic strength and 25 °C against the effective ionic radii of these elements in 8-fold coordination (taken from Shannon [15]) is shown in Fig. 5.

As shown in Fig. 5, the fit is good for all alkaline-earth metals which likely indicates that the bonding between these alkaline-earth metals and EDTA^4−^ is similar and relativistic or other effects do not occur. It also confirms that the electrostatic model is a useful tool for extrapolation of radium thermodynamic properties and obtaining a first estimate of stability constants for radium complexation.

### SIT Ion Interaction Parameters of Ba^2+^ and Ra^2+^

According to the SIT model (Eq. 5), the slopes are equal to the ion interaction parameters between oppositely charged ions. The slopes for the extrapolation to zero ionic strength in Figs. 1 and 3 yield the SIT ion interaction parameter terms shown in Eqs. 16, 17, 18, and 19, respectively:

16 $$ \Delta \varepsilon_{1}^{\text{BaEDTA}} = \varepsilon ({\text{Na}}^{ + } ,{\text{BaEDTA}}^{2 - } ) - \varepsilon ({\text{Ba}}^{2 + } ,{\text{Cl}}^{ - } ) - \varepsilon ({\text{Na}}^{ + } ,{\text{EDTA}}^{4 - } ) $$

17 $$ \Delta \varepsilon_{1}^{\text{RaEDTA}} = \varepsilon ({\text{Na}}^{ + } ,{\text{RaEDTA}}^{2 - } ) - \varepsilon ({\text{Ra}}^{2 + } ,{\text{Cl}}^{ - } ) - \varepsilon ({\text{Na}}^{ + } ,{\text{EDTA}}^{4 - } ) $$

18 $$ \Delta \varepsilon_{2}^{\text{BaEDTA}} = \varepsilon ({\text{Na}}^{ + } ,{\text{BaEDTA}}^{2 - } ) + \varepsilon ({\text{H}}^{ + } ,{\text{Cl}}^{ - } ) - \varepsilon ({\text{Ba}}^{2 + } ,{\text{Cl}}^{ - } ) - \varepsilon ({\text{Na}}^{ + } ,{\text{HEDTA}}^{3 - } ) $$

19 $$ \Delta \varepsilon_{2}^{\text{RaEDTA}} = \varepsilon ({\text{Na}}^{ + } ,{\text{RaEDTA}}^{2 - } ) + \varepsilon ({\text{H}}^{ + } ,{\text{Cl}}^{ - } ) - \varepsilon ({\text{Ra}}^{2 + } ,{\text{Cl}}^{ - } ) - \varepsilon ({\text{Na}}^{ + } ,{\text{HEDTA}}^{3 - } ) $$

The SIT ion interaction parameters determined for Eqs. 16–19 and some other ion interactions relevant to the studied systems are listed in Table 6.

**Table 6:** SIT ion interaction parameters kg·mol^−1^ of some metal ions and ligands relevant to the studied systems at 25 °C

Interaction	SIT parameters (kg·mol^−1^)	References
Δ*ε*_1_(MgEDTA^2−^)	−(0.52 ± 0.04)	[26]
Δ*ε*_1_(CaEDTA^2−^)	−(0.5 ± 0.5)	[26]
Δ*ε*_1_(BaEDTA^2−^)	−(0.44 ± 0.07)	Equation 16 (this work)
Δ*ε*_1_(RaEDTA^2−^)	−(0.54 ± 0.06)	Equation 17 (this work)
Δ*ε*_2_(BaEDTA^2−^)	0.14 ± 0.08	Equation 18 (this work)
Δ*ε*_2_(RaEDTA^2−^)	0.10 ± 0.07	Equation 19 (this work)
*ε*(Na^+^, EDTA^4−^)	0.32 ± 0.14	[25]
*ε*(Na^+^, HEDTA^3−^)	−(0.10 ± 0.14)	[25]
*ε*(H^+^, Cl^−^)	0.12 ± 0.01	[25]
*ε*(Ba^2+^, Cl^−^)	0.07 ± 0.01	[25]
*ε*(Na^+^, MgEDTA^2−^)	−(0.01 ± 0.15)	[25]
*ε*(Na^+^, BaEDTA^2−^)	−(0.03 ± 0.11)	This work
*ε*(Na^+^, RaEDTA^2−^)	−(0.10 ± 0.11)^a^	This work

Uncertainties correspond to 95% confidence interval

^a^This value has been calculated using *ε*(Ba^2+^, Cl^−^) as a substitute for *ε*(Ra^2+^, Cl^−^)

As shown in Table 6, the SIT parameters for all of the listed alkaline-earth metal ions are very similar. According to the SIT, interactions occur only between ions of opposite charge, which means that the alkaline-earth metal ions undergo similar short- and long-range electrostatic interactions with EDTA^4−^ and Cl^−^. The SIT ion interaction parameters between Na^+^ and BaEDTA^2−^ can be calculated as a weighted mean (Eqs. 16 and 18) and using the derived Δ*ε*_1_(BaEDTA^2−^) or Δ*ε*_2_(BaEDTA^2−^) and previously established ion interaction parameters: *ε*(Ba^2+^, Cl^−^), *ε*(H^+^, Cl^−^), *ε*(Na^+^, EDTA^4−^) and *ε*(Na^+^, HEDTA^3−^) [25]. The SIT ion interaction parameters between Na^+^ and RaEDTA^2−^ can be calculated using the same method, with *ε*(Ba^2+^, Cl^−^) continuing to substitute for *ε*(Ra^2+^, Cl^−^). All parameters are listed in Table 6 and a comparison of the computed *ε*(Na^+^, BaEDTA^2−^) and *ε*(Na^+^, RaEDTA^2−^) parameters with *ε*(Na^+^, MgEDTA^2−^), taken from the literature [25], shows that all parameters are within the 95% confidence intervals.

The barium ion interaction parameters are often used as a substitute for the radium parameters due to a lack of experimental data in the case of radium [5, 16, 17]. It is possible to verify this methodology by calculation of Δ*ε*_1_(RaEDTA^2−^) or Δ*ε*_2_(RaEDTA^2−^) (Eqs. 17 and 19) using *ε*(Na^+^, EDTA^4−^), *ε*(Na^+^, HEDTA^3−^) and the barium SIT parameters listed in Table 6 as substitutes for unknown radium parameters (i.e., *ε*(Na^+^, BaEDTA^2−^) instead of *ε*(Na^+^, RaEDTA^2−^) and *ε*(Ba^2+^, Cl^−^) instead of *ε*(Ra^2+^, Cl^−^)). This results in Δ*ε*_1_(RaEDTA^2−^) = −(0.42 ± 0.18) and Δ*ε*_2_(RaEDTA^2−^) = −(0.08 ± 0.18) which are within the 95% confidence intervals of the experimentally determined Δ*ε*_1_(RaEDTA^2−^) and Δ*ε*_2_(RaEDTA^2−^) SIT parameters. This indicates that the method of using the barium SIT parameters as a substitute for those of radium is valid for the Ra^2+^–NaCl–EDTA^4−^ system at ionic strengths below 3.5 mol·kg^−1^.

## Conclusion

The apparent stability constants of the BaEDTA^2−^ and RaEDTA^2−^ complexes were determined over a wide range of NaCl concentrations (0.2–2.5 mol·L^−1^) at 25 °C and in two pH regions where the EDTA^4−^ and HEDTA^3−^ species dominate. The obtained constants were extrapolated to zero ionic strength using the SIT and compared with available literature data. It was found that in the pH region where the HEDTA^3−^ species dominates, the reaction of Ba^2+^ or Ra^2+^ with the HEDTA^3−^ ligand results in the formation of the BaEDTA^2−^ and RaEDTA^2−^ complexes and a proton release and that formation of BaHEDTA^−^ or RaHEDTA^−^ does not occur in alkaline media. The similarity of the barium and radium ion interaction parameters indicates that both metal ions undergo almost identical short- and long-range electrostatic interactions with EDTA^4−^ and Cl^−^. The results also show that using the SIT interaction parameters of Ba^2+^ as a substitute for missing Ra^2+^ SIT interaction parameters is a useful tool for the Ra^2+^–NaCl–EDTA^4−^ system.
